# The Rapid Online Cognitive Assessment for the Detection of Neurocognitive Disorder: Open-Label Study

**DOI:** 10.2196/66735

**Published:** 2025-06-19

**Authors:** Calvin Howard, Amy Johnson, Joseph Peedicail, Marcus C Ng

**Affiliations:** 1 Department of Neurology, Brigham and Women's Hospital, Harvard Medical School, 60 Fenwood Road 1st Floor, Boston, MA, 02115, United States; 2 Section of Neurology, Department of Internal Medicine, University of Manitoba, Winnipeg, MB, R3T 2N2, Canada; 3 Klinik für Neurologie mit Experimenteller Neurologie, Charité – Universitätsmedizin Berlin, Berlin, Germany; 4 Graduate Program in Biomedical Engineering, University of Manitoba, Winnipeg, MB, Canada

**Keywords:** cognitive, neurology, dementia, geriatrics, artificial intelligence, assessment, online assessment, cognitive assessment, cognition, screening, remote screening, cognitive examination, Rapid Online Cognitive Assessment, RoCA, cohort

## Abstract

**Background:**

The rising prevalence of dementia necessitates a scalable solution to cognitive screening. Paper-based cognitive screening examinations are well-validated but minimally scalable. If a digital cognitive screening examination could replicate paper-based screening, it may improve scalability while potentially maintaining the performance of these well-validated paper-based tests. Here, we evaluate the Rapid Online Cognitive Assessment (RoCA), a remote and self-administered digital cognitive screening examination.

**Objective:**

The objective of this study was to validate the ability of RoCA to reliably evaluate patient input, identify patients with cognitive impairment relative to the established tests, and evaluate its potential as a screening tool.

**Methods:**

RoCA uses a convolutional neural network to evaluate a patient’s ability to perform common cognitive screening tasks: wireframe diagram copying and clock drawing tests. To evaluate RoCA, we compared its evaluations with those of established paper-based tests. This open-label study consists of 46 patients (age range 33-82 years) who were enrolled from neurology clinics. Patients completed the RoCA screening examination and either Addenbrooke’s Cognitive Examination-3 (ACE-3, n=35) or Montreal Cognitive Assessment (MoCA, n=11). We evaluated 3 primary metrics of RoCA’s performance: (1) ability to correctly evaluate patient inputs, (2) ability to identify patients with cognitive impairment compared to ACE-3 and MoCA, and (3) performance as a screening tool.

**Results:**

RoCA classifies patients similarly to gold standard paper-based tests, with a receiver operating characteristic area under the curve of 0.81 (95% CI 0.67‐0.91; *P*<.001). RoCA achieved sensitivity of 0.94 (95% CI 0.80‐1.0; *P*<.001). This was robust to multiple control analyses. Approximately 83% (16/19) of the patient respondents reported RoCA as highly intuitive, with 95% (18/19) perceiving it as adding value to their care.

**Conclusions:**

RoCA may act as a simple and highly scalable digital cognitive screening examination. However, due to the limitations of this study, further work is required to evaluate the ability of RoCA to be generalizable across patient populations, assess its performance in an entirely remote manner, and analyze the effect of digital literacy.

## Introduction

Current projections estimate 150 million patients with dementia worldwide by 2050, with 57 million as of 2019 [1]. This causes considerable health care system strain, leaving a majority of patients undiagnosed [2-5]. However, for the patients who do receive a diagnosis, it often takes 3 years or longer from symptom onset [2,3,6-8]. The next step, that is, receiving an etiological diagnosis like Alzheimer disease, requires even more time [4,8].

Much research has previously focused on evaluating the contributors to these problems [9-13]. Frontline physicians report two key addressable issues: (1) the logistical difficulty of screening enough patients [9,10] and (2) variable comfort in diagnosing patients with dementia [6,14].

Digital cognitive assessments (DCAs) offer a promising solution to these issues [15-17]. They provide high scalability, which addresses logistical difficulties, and can render expert-level diagnoses, which address the issue of diagnostic comfort. However, most DCAs have limitations preventing them from completely addressing these two issues [18]. First and foremost is that these novel tests lack validation [15]. DCAs often use completely new testing methods, diverging from the well-validated methods that made paper-based tests so valuable [15-17,19,20]. The extensively validated nature of these tests helps define the patient populations and use-cases for these tests, while the creation of novel unrelated tests requires repetition of this process. It may be possible to piggyback new-age cognitive tests upon established tests, helping them achieve the generalizability that required decades of work and iteration to accomplish with established tests. Second, poor design choices often reduce accessibility [16,17,21-25]. Common examples are burying test access deep within websites, requiring users to make accounts, or having patients use unfamiliar hardware. Lastly, most DCAs are not truly scalable [16,17,21-25]. Despite being digital, they often require specific tablets, computers, on-site testing, or even expert test evaluators—these choices bottleneck patient access [19,26,27].

The Rapid Online Cognitive Assessment (RoCA) is a DCA that aims to address these limitations. To stay consistent with well-validated methods, it reproduces the screening results of gold-standard paper-based tests: Addenbrooke’s Cognitive Examination-3 (ACE-3) and the Montreal Cognitive Assessment (MoCA) [28-31]. Patient input directly guided its design to ensure accessibility, resulting in a short touchscreen-based drawing battery with automated convolutional neural network–based scoring [32,33]. For scalability, RoCA is entirely automated, remote, functions on all devices, and utilizes cloud computing to enhance geographic access. Finally, RoCA aims to specifically act as a screening examination. For this reason, we prioritized its sensitivity.

We first ensured that RoCA’s underlying machine learning works well by assessing the accuracy of its neural network. Then, we compared RoCA’s similarity to gold standard paper-based tests and evaluated its accessibility computationally and with patient input. Lastly, we provide the data-driven thresholds that maximize its sensitivity, optimizing its function as a screening examination [34,35].

## Methods

### Ethical Considerations

This study was conducted in accordance with ethical standards as laid down in the 1964 Declaration of Helsinki and its later amendments. Approval was received from the Research Ethics Board of the Bannatyne Campus, University of Manitoba (#HS25666). Patients were recruited from neurology and neuropsychology clinics, and all signed institutional review board–approved consent forms. Substitute decision makers were included in the consent process of patients with cognitive impairment. Patients did not receive compensation for involvement in the study. This work was conducted between October 01, 2022 and December 01, 2024.

### Study Participants

Our study cohort enrolled patients from neurology clinics across the Health Sciences Center, University of Manitoba (n=46). Patients with and without cognitive impairment were recruited. The inclusion criterion was English fluency. The exclusion criteria were acute psychiatric disorder contributing to cognitive state, disability restricting ability to utilize screens, disability restricting ability to receive visual and auditory instructions, developmental delay, acute medical condition contributing to cognitive state, and specifically delirium. Patients indicating interest in clinical research were contacted by study team members via phone.

Interested patients were screened for inclusion and exclusion criteria and enrolled. At the first clinic visit, patients were again screened for inclusion or exclusion criteria by a physician. Patients or their caregivers provided written consent at the first clinic visit. Patients were recruited until sample size for statistical power was achieved.

### Study Design

Patients were tested in a quiet environment by a physician trained in cognitive examination. RoCA was completed on a touchscreen tablet. RoCA automatically administered instructions to the patient and was completed automatically without interference or prompting from the examiner. The responses were automatically scored and summated without staff involvement. During RoCA, the patient was observed, but there was no interference from study staff in evaluation or scoring. ACE-3 and MoCA were administered and scored according to standard guidelines by one of the 3 trained experts [30,31]. Caregivers were allowed to join but could not participate in the examination.

### Cognitive Status Classification

A trained clinician administered a label of cognitive impairment based on the established cutoffs for each test: 26/30 on MoCA and 83/100 on ACE-3 [30,31,36].

### Sample Size Calculation

Patients were enrolled based on sample size requirements defined by the Hanley and McNeil formula [37]. This formula is based on the area under the curve (AUC) of the receiver operating characteristic and describes how sample size requirements vary with AUC. Under relatively good performance, an AUC of 0.70 and 80% statistical power is achieved with 16 positive and 16 negative cases. Under optimal performance with an AUC of 0.90, 80% statistical power is achieved with 2 positive and 2 negative cases. We conservatively aimed to recruit 16 participants with cognitive impairment and 16 without cognitive impairment.

### RoCA Cognitive Screening Examination

RoCA is a self-administering cognitive screening examination, which is compatible with smartphones, tablets, and personal computers. It relies upon devices having an internet connection to ensure all patients can access it, regardless of hardware specifications or specific device.

RoCA consists of 3 questions. Similar to previously described batteries, patients are asked to copy a line diagram of a cube, copy overlapping infinities, and perform a clock drawing [32]. For each question, the patient has an unrestricted amount of time to answer. Each question’s instructions are provided via a closed-captioned audio. Instructions may be repeated up to 3 times, but no further assistance is provided. Questions are answered via a touchscreen, although a keyboard and mouse may be used. A correct cube drawing is worth 2 points, overlapping infinities 1 point, and a clock 5 points. Incorrect drawings are worth 0 points. The total possible score is 8 points.

### RoCA Deployment

**Figure 1.**
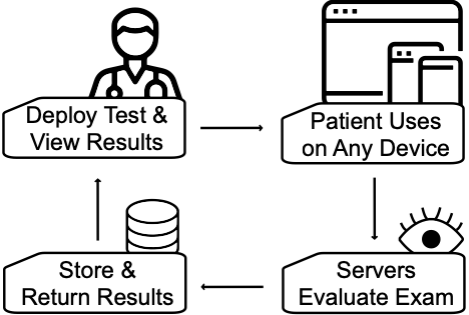
The Rapid Online Cognitive Assessment (RoCA) deployment system. Clinicians begin the deployment system. They can use the administrative platform to deploy an access link to patients. Patients subsequently receive the access link and are then able to take the interactive test on any device via the internet. Once the patient completes the examination, their answers are passed to the servers, which then evaluate the patient’s RoCA. The results are then stored in a database and are available for the patient’s clinician to see. Clinicians can view a patient’s results from the administrative platform.

The RoCA evaluation starts with the clinician (Figure 1). The clinician uses an administrative platform, from which they send tests and view results, to send an encrypted access link to a patient. Patients open this link to begin RoCA. Links are specific to each given patient and are inactivated after use. Upon completing the test, the results are encrypted and sent to a scoring server on a private subnet. The scoring server sends encrypted scores to an encrypted database on a private subnet. The administrative platform receives scores from this database, allowing the clinician to view the results upon completion. The system is Health Information Privacy Protection Act–compliant.

### Patient Drawing Classification

**Figure 2.**
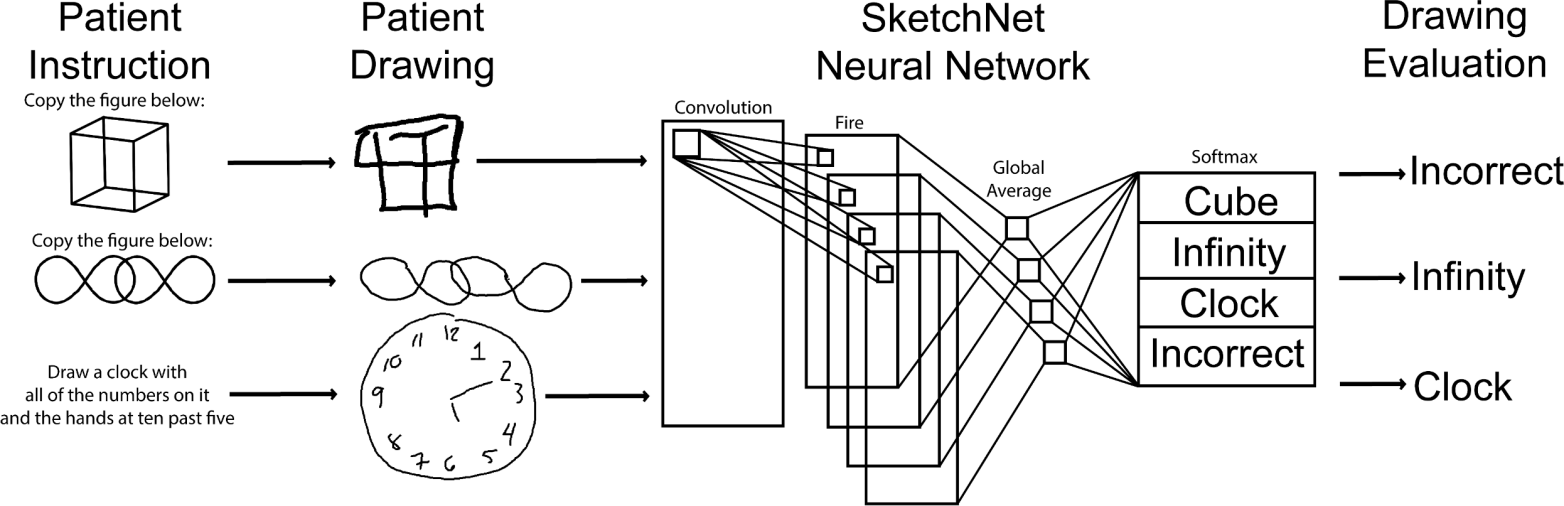
The Rapid Online Cognitive Assessment (RoCA) drawing evaluation system. Patients receive audiovisual instructions asking them to draw 3 different images. The first 2 tasks are image-copying tasks of a wire cube and overlapping infinities. The last question is to draw a clock face at 10 minutes past 5 PM. The patient drawings are then retrieved and preprocessed. After preprocessing, the images are then passed to SketchNet, a convolutional neural network, to classify the images. The output of SketchNet is the classification of the input image. The SketchNet is then applied to each image in RoCA.

Patient drawings were evaluated using SketchNet, a convolutional neural network built specifically to evaluate RoCA inputs (Figure 2) [33,38]. Briefly, the SketchNet is a convolutional neural network using a SqueezeNet architecture [39]. This particular architecture is composed of convolutional layers, fire modules, and finishes with a global average and softmax. This allows high degrees of accuracy while maintaining the speed of classifications and a small overall size of the model. It was trained using transfer learning, pretrained on ImageNet, and subsequently trained on thousands of RoCA-specific drawings to evaluate cognitive test drawings with 97% accuracy [38,39].

SketchNet-based classifications were compared to ground truth: assessment of drawings by a clinician trained in cognitive examinations. The drawings were scored according to established scoring guidelines [30].

### Evaluation of Drawing Classification

The SketchNet is inherently a multiclass classifier, which yields complex classification evaluations. To simplify this, we condensed classifications into correct versus incorrect. Confusion matrices were constructed from SketchNet classification outputs. Using the confusion matrix, we derived all classification metrics: accuracy, sensitivity, specificity, positive predictive value, and negative predictive value. This was repeated for each drawing.

### Comparison of Drawing Classification Against a Statistical Baseline

We next investigated if RoCA was classifying images better than that expected by chance. To do this, we developed a random classifier, which represents classification performance at chance level. It is calculated by deriving a confusion matrix (true positives, true negatives, false positives, false negatives) under chance circumstances. The confusion matrix is thus the probability of selecting a given class multiplied by the probability of an image being a given class (equation S1 in Multimedia Appendix 1). All classification metrics then follow from this confusion matrix. These provide the chance-level baseline.

To compare the classification of each drawing, we bootstrapped SketchNet classifications (n=1000) and counted the number of times the bootstrap fell below chance level. This is the *P* value. To compare the overall performance, we averaged performance across RoCA and the chance-level performance and then compared them with an independent 2-sided *t* test.

### Evaluation of Diagnostic Classification

To evaluate the reliability of RoCA patient classifications, we compared RoCA classification to ground-truth classification. Ground-truth classification is the classification adjudicated by ACE-3 or MoCA in accordance with the established guidelines [30,31,36]. A receiver operating characteristic was constructed, and its AUC was calculated to measure diagnostic performance. The Youden Index was calculated to derive the optimal classification threshold [40]. Patient classifications were based upon this threshold, and these were then used to construct confusion matrices for RoCA.

### Statistical Evaluation of RoCA Classification

To derive a statistical baseline for RoCA, we again employed a random classifier equivalent to RoCA. The confusion matrix was again generated using probabilities of selecting each class, as described above (equation S1 in Multimedia Appendix 1). The random-chance baseline for the AUC was chosen to be 0.50, in accordance with the literature [37]. To compare RoCA to these chance-level baselines, we again used the bootstrapping technique described above (n=10,000).

### Classification Confidence

To evaluate the confidence of RoCA classifications, we estimated this directly with the confidence intervals derived from bootstrapping (n=10,000) [41,42]. At all possible RoCA diagnostic thresholds, we derived the confusion matrices and classification metrics for each bootstrap. This allows the observation of RoCA confidence across all possible thresholds. To make sure that an appropriate threshold is chosen to optimize RoCA for a screening examination, we focused on evaluating the sensitivity and negative predictive value across all thresholds.

### Covariates Influencing the RoCA Score

We aimed to identify the clinical covariates that might be influencing RoCA scores. To do this, we collected several covariates: age, ethnicity, sex, educational status, employment status, and which paper-based examination they received. These covariates were then related to RoCA scores. This was done using a multivariate regression of all variables upon the RoCA score.

We also assessed if any individual covariate compounded the effect of impaired cognition. This was done with a series of additional regressions. In these, the covariate, cognitive status, and their interactions were regressed upon the RoCA score. This was done for each covariate.

### RoCA Usability and Patient Perception

A follow-up survey was sent to patients within 6 months of completing RoCA. Patients responded to a battery of questions by using both dichotomous (yes or no) questions and Likert scale questions. Likert scales were adjudicated such that 1 corresponded to very low, 2 was low, 3 was moderate, 4 was high, and 5 was very high.

### Statistical Analysis

All analyses were performed in Python. The 2-sided *t* testing was performed with SciPy [43]. Scikit-learn was used for receiver operating characteristic curve construction [44]. Regression analyses were performed with statsmodels and an ordinary least squares regression [45]. Confidence intervals were calculated using bootstrapping with replacement (n=1000) [41].

## Results

### Patient Characteristics

In this study, 143 patients were assessed for eligibility; 79 patients declined enrollment, 7 did not meet the inclusion criteria, 57 were enrolled, and 11 did not make their appointment. Finally, 46 patients completed the study. There were 16 patients with cognitive impairment and 30 patients without cognitive impairment. Patient demographics are shown in Table 1.

**Table 1.** Patient demographics (N=46).

Parameter	Values
Age (years), mean (SD)	49.1 (15.0)
Sex, n (%)	
Female	24 (52)
Male	22 (48)
Educational status, n (%)	
Less than secondary	2 (4)
Postsecondary	8 (17)
Secondary	36 (78)
Employment status, n (%)	
Employed	29 (63)
Unemployed	17 (37)
Ethnicity, n (%)	
African	1 (2)
Caucasian	31 (67)
European	1 (2)
Filipino	3 (7)
Indian	4 (9)
Indigenous	6 (13)
Cognitive status, n (%)	
Impaired	17 (37)
Intact	29 (63)
Diagnosis, n (%)	
Neurologically healthy	16 (35)
Mild cognitive impairment	9 (20)
Probable Alzheimer disease	7 (15)
Epilepsy	14 (30)
Cognitive examination, n (%)	
ACE-3^a^	35 (76)
MoCA^b^	11 (24)

a ACE-3: Addenbrooke’s Cognitive Examination-3.

b MoCA: Montreal Cognitive Assessment.

### RoCA’s Evaluation of Patient Drawings

**Figure 3.**
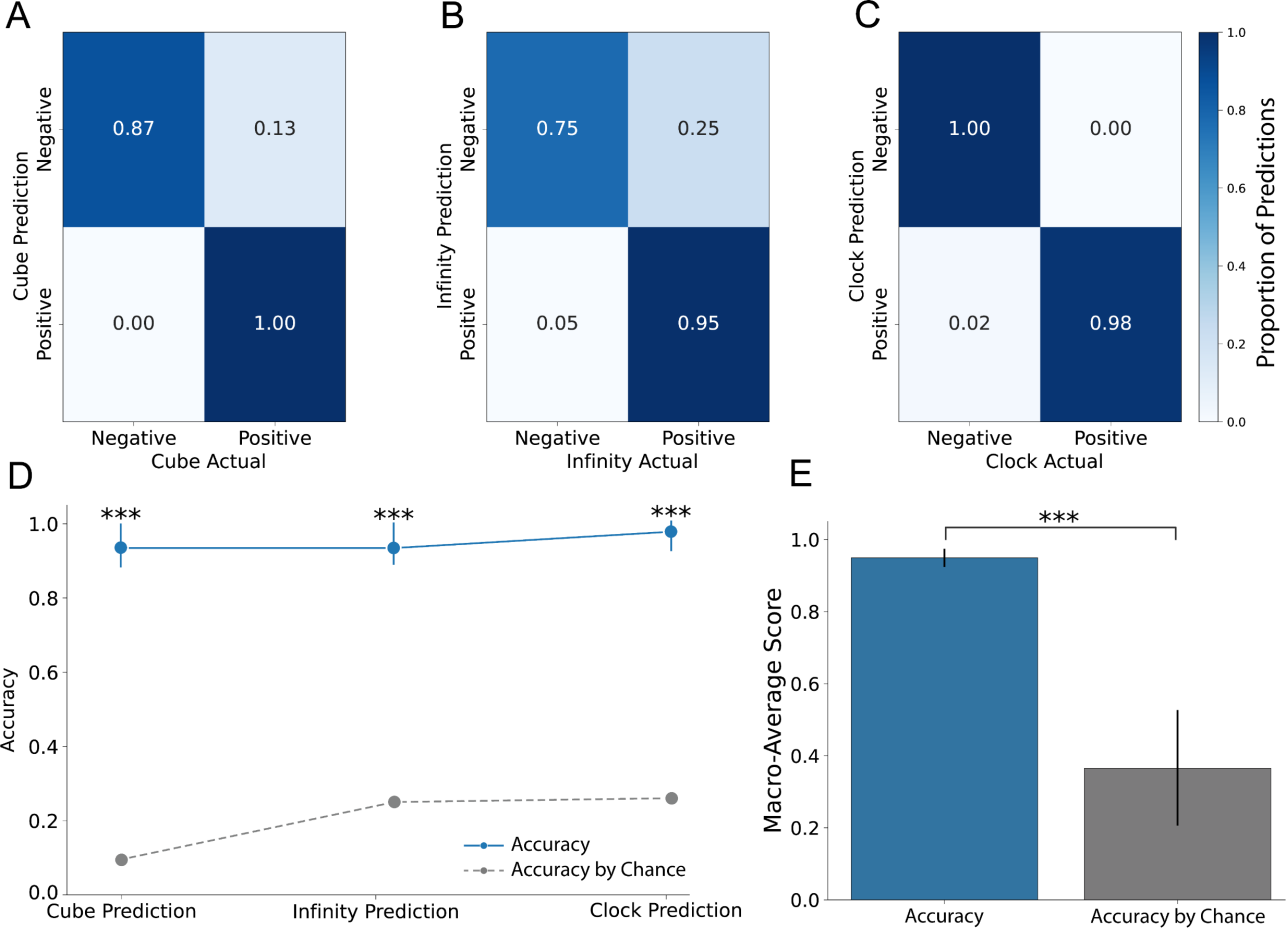
Accurate classification of patient drawings by the Rapid Online Cognitive Assessment (RoCA). (A) Confusion matrix for cube classification. (B) Confusion matrix for infinity classification. (C) Confusion matrix for clock classification. (D) Accuracy of RoCA compared to that expected by chance for all drawings. Bootstrapped confidence intervals were used to statistically compare the observed accuracy of RoCA to that expected by chance. (E) The overall accuracy of RoCA is significantly higher than that expected by chance.

We first evaluated how RoCA evaluated patient drawings (Figure 3A-C). RoCA classified 97% (44/46) of the cubes correctly, 91% (42/46) of the infinities correctly, and 98% (45/46) of the clocks correctly. We next calculated the accuracy of RoCA for each drawing individually (Figure 3D). We compared the accuracy of each drawing to its statistical baseline by bootstrapping, resampling the accuracy, and counting the number of times it fell below the random classifier. The accuracy for the cube was 93% (95% CI 0.85‐1.0; *P*<.001), for the overlapping infinities was 94% (95% CI 0.87‐1.0; *P*<.001), and for the clock was 98% (95% CI 0.90‐1.0; *P*<.001). Finally, we derived the overall accuracy of RoCA across all drawings (Figure 3E). RoCA had 95% (SD 3%) accuracy across all drawings, which was higher than that expected by chance (*P*<.001). Additional classification metrics are available in Table S1 of Multimedia Appendix 1.

### RoCA Achieves Similar Diagnostic Fidelity to Gold-Standard Tests

**Figure 4.**
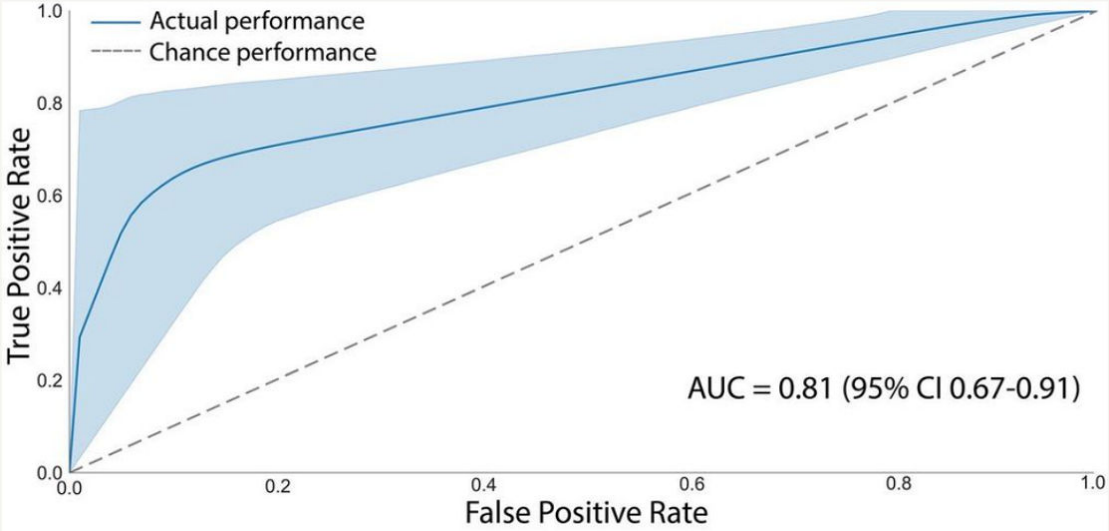
Diagnostic performance of the Rapid Online Cognitive Assessment (RoCA) compared to that of the gold-standard paper-based test. The receiver operating characteristic of RoCA achieved a value of 0.81 (95% CI 0.67‐0.91; *P*<.001). The receiver operating characteristic curve was calculated by bootstrapping RoCA’s overall classification to derive confidence intervals and mean performance. The AUC presented is the mean AUC across all bootstraps. The shaded region represents 95% CI. The point-estimate AUC or the AUC without any bootstrap is 0.85. AUC: area under the curve.

Next, we compared the overall performance of RoCA to that of the gold-standard tests (Figure 4). Each patient’s RoCA score was related to their diagnostic classification by using a receiver operating characteristic curve compared to ACE-3 and MoCA classifications. The AUC was 0.81, which was significantly higher than that expected by chance (95% CI 0.67‐0.91; *P*<.001). RoCA was found to outperform patient classification expected by chance (*χ*^2^_1_=1.7; *P*<.001). This was robust, regardless of whether ACE-3 (AUC=0.79, 95% CI 0.67‐0.86; *P*<.001) or MoCA (AUC=1.0, 95% CI 1.0‐1.0; *P*<.001) was used. Subsequently, we found the optimal threshold for RoCA was 7/8, according to Youden Index [46].

### Screening Performance of RoCA

We next evaluated RoCA’s ability to act as a screening examination. To do this, we evaluated RoCA’s accuracy, sensitivity, and negative predictive value. We began by developing a random classifier equivalent of RoCA, which we used to derive the RoCA’s statistical baseline for comparison (Figure S1 in Multimedia Appendix 1). All screening metrics were expected to be low by chance, with an expected accuracy of 50%, sensitivity of 50%, and negative predictive value of 63%.

**Figure 5.**
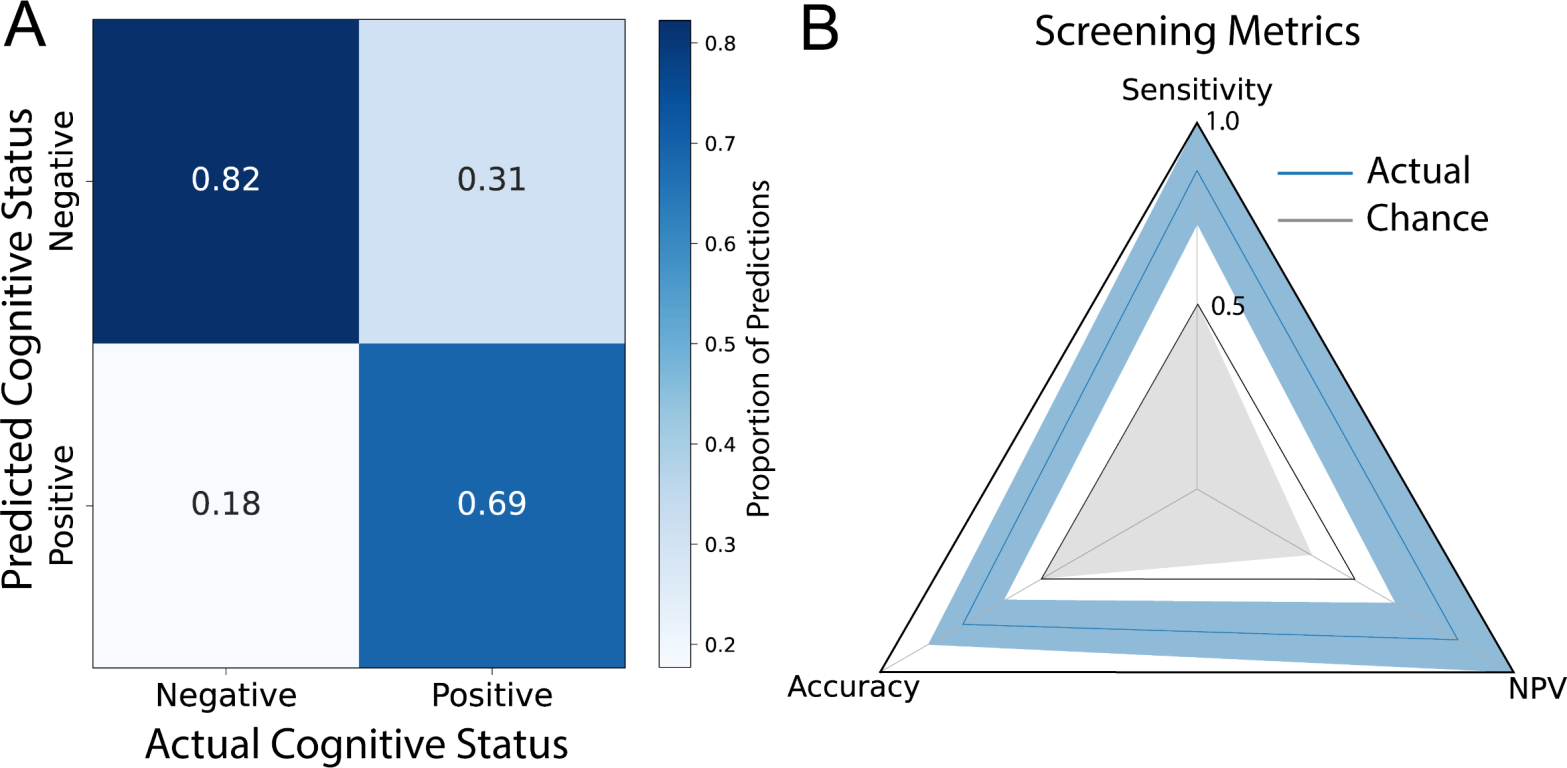
Screening performance of the Rapid Online Cognitive Assessment (RoCA). (A) Confusion matrix for the classification of RoCA at the threshold, normalized by predictions. The true positive rate was 62% (16/26), true negative rate was 95% (19/20), false negative rate was 5% (1/20), and false positive rate was 38% (10/26). (B) Screening metrics of RoCA presented in a radar plot. The solid blue vertices represent the measured screening metric, with shaded blue edges marking the 95% CIs. The gray interior represents the expected performance by random chance. RoCA achieved an excellent sensitivity of 0.94 (95% CI 0.63‐0.89; *P*<.001) and an excellent negative predictive value of 0.95 (95% CI 0.84‐1.0; *P*<.001). Accuracy is also presented, although it is not purely a screening metric, and it was better than that expected by chance at 0.76 (95% CI 0.63‐0.89; *P*<.001). NPV: negative predictive value.

Following this, we evaluated the actual RoCA’s screening performance. We began by calculating the confusion matrix of the actual RoCA by using the optimal threshold of 7/8 (Figure 5A). We then calculated the screening metrics for RoCA (Figure 5B). At the optimal threshold, RoCA has an accuracy of 0.76 (95% CI 0.63‐0.89; *P*<.001), which was better than that expected by chance. It also achieved both superior and statistically significant sensitivity of 0.94 (95% CI 0.81‐1.0; *P*<.001) and a statistically significant negative predictive value of 0.95 (95% CI 0.84‐1.0; *P*<.001). Specificity and positive predictive value were also calculated for completeness, although they are not directly related to the screening ability (Table S2 in Multimedia Appendix 1).

### Diagnostic Confidence of RoCA

**Figure 6.**
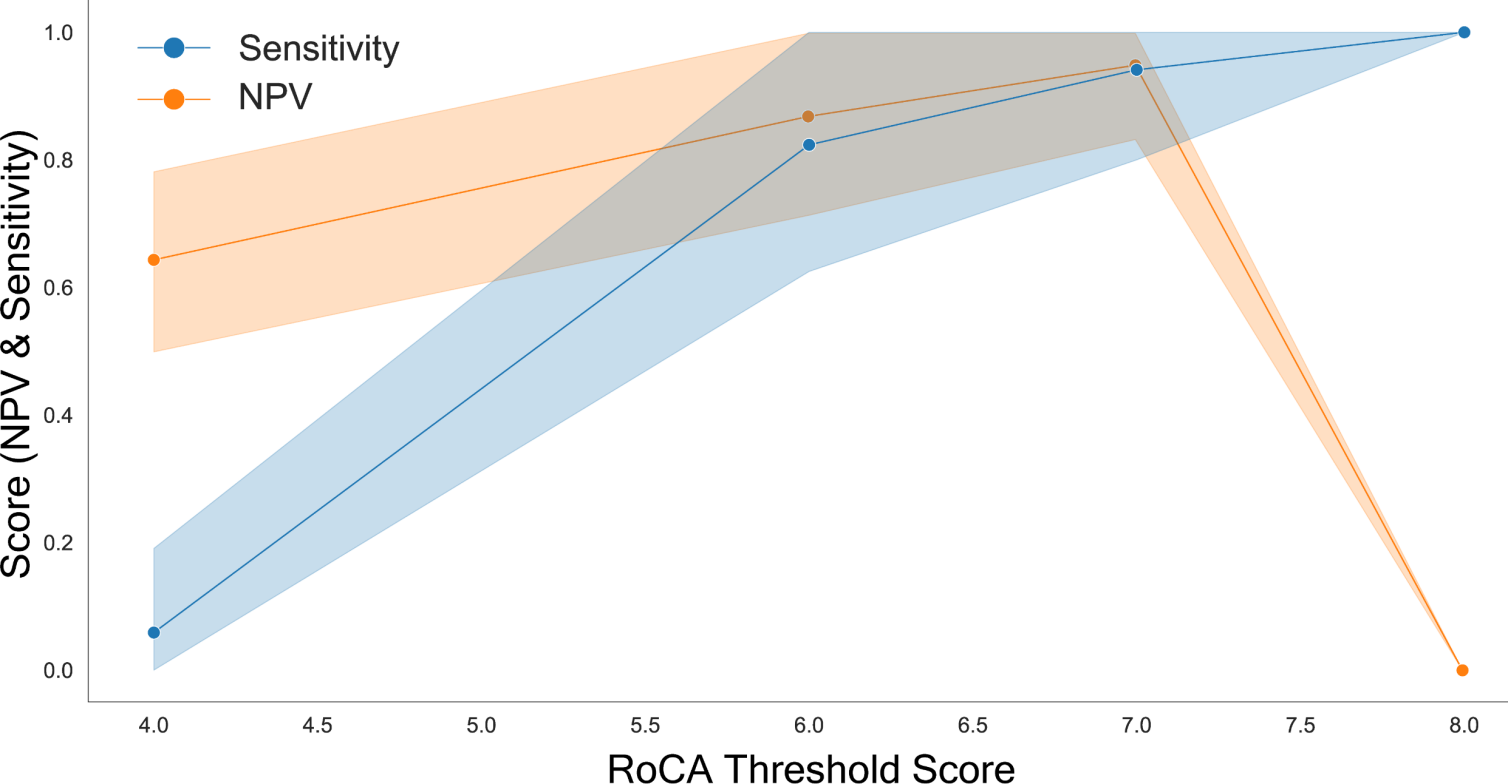
At the optimal threshold of 7/8, RoCA optimizes both sensitivity and NPV. At 7/8, the values of sensitivity and NPV are maximized with minimization of their uncertainty. At this point, sensitivity is also highly confident (sensitivity=0.94, 95% CI 0.80‐1.0). NPV is also maximized (0.80, 95% CI 0.83‐0.95). Shaded regions represent 95% CIs derived from bootstrapping (n=10,000). Points represent the estimated sensitivity and NPV without bootstrapping. NPV: negative predictive value. RoCA: Rapid Online Cognitive Assessment.

Next, we ensured that the chosen RoCA threshold is the optimal threshold for screening. Youden J, calculated using ROC, balances sensitivity and specificity, and therefore will not necessarily result in the optimal screening threshold. To search for the optimal screening threshold, we calculated the confidence of sensitivity and negative predictive value across all potential scores (Figure 6). We found that the threshold of 7/8, identified by AUC, was the optimal screening threshold. It simultaneously maximized the sensitivity (0.94) and negative predictive value (0.95) while also optimizing the confidence interval for sensitivity (95% CI 0.81‐1.0) and negative predictive value (95% CI 0.84‐1.0). Specificity and positive predictive value were also calculated for completeness and are available, although these are not optimized in screening examinations (Figure S2 in Multimedia Appendix 1).

### Influence of Patient Demographics on RoCA Score

We next evaluated whether any patient factors may be influencing RoCA performance. We first performed one multivariate regression to evaluate the relationship of all the demographic variables with the RoCA score (Figure S2 in Multimedia Appendix 1). However, only cognitive status had a significant association with the RoCA score (β=1.07; *P*<.001). No other patient factor was related. We also performed a series of multivariate regressions for each covariate, assessing if patient factors might compound the effects of impaired cognition (Figure S3 in Multimedia Appendix 1). Again, we found that only cognitive status was significantly associated with RoCA scores. Lastly, we evaluated the time investment required to complete RoCA. We found that RoCA takes roughly 2 minutes and 30 seconds (mean 148, SD 34 s).

### Patient Perspective of RoCA

**Figure 7.**
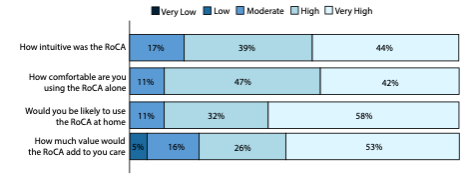
Patients’ accessibility of RoCA. This is a bar plot of the survey responses to Likert scale questions from 19 patients post RoCA. RoCA: Rapid Online Cognitive Assessment.

Finally, we evaluated accessibility as reported by patients. We did this using post-RoCA survey responses, wherein patients answered questions specifically regarding the accessibility of RoCA (Figure 7); 19 patients (mean age 60.2, SD 18 years) of the 46 patients responded. The cognitive status of the respondents was unknown, given the anonymous nature of the survey.

Likert scales were used to derive an evaluation of the overall patient evaluation of RoCA. Approximately 83% (16/19) of the respondents reported RoCA as highly or very highly intuitive, 90% (17/19) of the respondents reported high or very high comfort in using RoCA, 90% (17/19) of the respondents reported they would be highly or very highly confident using the test alone, and 79% (15/19) of the respondents reported that RoCA would add a high or very high degree of value to their medical care.

We also asked patients a series of yes or no questions regarding other aspects of RoCA (Table S3 in Multimedia Appendix 1). Among them, we found 100% of the patients would want to take the test prior to appointments to discuss results, and 100% of the patients would trust RoCA’s results. Most surprisingly, we found 55% (10/19) of the patients made appreciable lifestyle changes after using RoCA. These changes specifically included starting cognitive exercises, beginning a dementia-friendly diet, starting physical exercise, or doing financial planning.

## Discussion

### Interpretation of Results

We found that RoCA performed well in this single-center open-label trial. RoCA can evaluate the drawings used in cognitive examinations and subsequently use them to sensitively screen for cognitive impairment. Importantly, RoCA does this in-line with established paper-based tests. Further, we found that RoCA is an accessible screening system, as we did not find significant effects of patient factors such as age or demographics. This combined with RoCA’s cloud-based platform allows it to act as a sensitive, accessible, and scalable digital cognitive screening examination.

### Role of RoCA in Screening

The first limitation of DCAs is their lack of validity compared to paper-based screening systems [15-17,19,20]. RoCA specifically aimed to classify patients similarly as paper-based screening systems, thereby acting as a digital surrogate for them [28-31,47-49]. We found that RoCA rules out (screens) cognitively healthy patients, which would have been similarly ruled out by standard paper-based tests.

The primary benefit of RoCA’s sensitivity is triaging patients for further examination. It can prioritize at-risk patients while offloading the cognitively healthy for routine observation. This process eliminates the proportion of true negatives from the population, who go on for subsequent assessments, thereby increasing the positive predictive values and negative predictive values of any further evaluation [50]. RoCA may benefit individual clinical practices in triaging patients, or it could aid large-scale screening of patient populations.

### RoCA in Context

Although the currently available list of digital cognitive examinations has been comprehensively reviewed elsewhere [15,16], we will briefly place RoCA in the context of other digital cognitive examinations. RoCA has several key distinguishing features. First, RoCA is entirely automated and self-administered, allowing it to be administered remotely. Other digital cognitive examinations such as MoCA Duo require an expert to physically administer the test [19], similar to the paper-based testing. Second, RoCA is developed for accessibility. Most digital cognitive tests require patients to navigate complex websites [21], app stores [51], or use keyboards and mouses [52]. RoCA is developed such that patients simply receive an access link via text or email, click it, and complete the test on any device of their choosing. Finally, RoCA specifically emulates established paper-based tests. Other DCAs often develop new machine learning algorithms or cognitive evaluation maneuvers [51-53] and then relate these to specific diagnostic classifications. However, although these are potentially highly useful, the generalizability of these new tests is unclear and will require extensive additional studies to demonstrate generalizability and validity similar to paper-based tests.

### Limitations

This study and RoCA are not without limitations. First, this study does not focus on individual etiologies causing dementia but focuses on identifying cognitive impairment as a whole. Thus, it is possible that etiologies presenting in different cognitive domains may result in variable RoCA performance. However, before specializing into the evaluation of different etiologies, it is critical to accurately screen cognitive impairment itself. This will help RoCA generalize across disorders causing cognitive impairment. Another limitation is the sample size. Although this study’s sample size was defined using a power analysis, RoCA will require additional testing in larger cohorts across additional demographics, larger age ranges, specific neurodegenerative etiologies, and a variety of digital literacy levels. Additionally, further studies will need to be performed with blinding. Lastly, the survey results must be interpreted with caution. Due to the nature of surveys and the 6-month survey time frame, it is possible that the survey results are subject to biases such as the selection bias.

There are limitations to RoCA. First, RoCA is hardware-dependent. For patients without touchscreens, they may have difficulty in generating high-quality drawings, which may hinder performance. However, RoCA was specifically trained on a dataset mixing drawings generated from touchscreens, mouses, and stylets to specifically offset this risk. Beyond this, RoCA is internet connection–dependent. However, we have ensured that this is compatible with smartphones to leverage their inherent internet connection. Although RoCA has been developed to be remotely administered and automatically scored, this study only evaluates the ability of a patient to perform RoCA alone, but still within the clinic. Thus, the robustness of RoCA under variable conditions in a fully remote setting must be evaluated in further works. It remains to be seen how stable RoCA scores are over time, and test-retest reliability must be assessed in future studies. Finally, RoCA is specifically a screening examination. It is not a test developed to have high specificity. Thus, to consolidate a positive screening result for a diagnosis of cognitive impairment, a patient should receive a subsequent evaluation with high specificity. For these reasons, RoCA is best used in a 2-part system, wherein the first test prioritizes sensitivity, while the second prioritizes specificity. For example, RoCA could be followed by our other full-length diagnostic test, the Autonomous Cognitive Examination [54,55]. The utility of combining these 2 tests at scale will require further work.

## Supplementary material

10.2196/66735 Multimedia Appendix 1 Supplementary data.

## Abbreviations

ACE-3: Addenbrooke’s Cognitive Examination-3
AUC: area under the curve
DCA: digital cognitive assessment
MoCA: Montreal Cognitive Assessment
RoCA: Rapid Online Cognitive Assessment
